# Fragile research systems, brain drain, and predatory publishing in under-resourced countries

**DOI:** 10.1093/femsml/uqag011

**Published:** 2026-03-24

**Authors:** Stipan Jonjić, Zeynep Ceren Karahan, Puri Lopez-Garcia, Paul Williams, Ani Gerbin, Luka Traven, Kenneth N Timmis, Paul B Rainey

**Affiliations:** Center for Proteomics, Faculty of Medicine, University of Rijeka, Rijeka 51000, Croatia; Croatian Academy of Sciences and Arts, Department of Biomedical Sciences in Rijeka, Rijeka 51000, Croatia; Department of Medical Microbiology and Ibn-i Sina Hospital Central Microbiology Laboratory, Ankara University School of Medicine, Ankara 06230, Turkey; Ecologie Societe Evolution, CNRS, Universite Paris-Saclay, AgroParisTech, Gif-sur-Yvette 91190, France; Biodiscovery Institute and School of Life Sciences, University of Nottingham, Nottingham NG72RD, United Kingdom; Center for Proteomics, Faculty of Medicine, University of Rijeka, Rijeka 51000, Croatia; Department of Environmental Medicine, Faculty of Medicine, University of Rijeka, Rijeka, 51000, Croatia; Institute of Microbiology, Technical University Braunschweig, Braunschweig 38106, Germany; Department of Microbial Population Biology, Max Planck Institute for Evolutionary Biology, Plon 24306, Germany & Laboratory of Biophysics and Evolution, CBI, ESPCI, Paris, Universite PSL, CNRS, Paris 75005, France

**Keywords:** fragile research systems, brain-drain, EU widening countries, predatory publishing practice, research culture

## Abstract

Many countries with lower research & innovation capacity face persistent constraints in building stable research systems. Chronic underfunding and weak science policy reduce institutional capacity and limit researchers' career prospects. These conditions encourage brain drain, particularly among early-career scientists who seek predictable funding, transparent evaluation, and merit-based advancement. As a result, research institutions lose skilled personnel, which weakens scientific training, governance, and research output. Additionally, within this environment, predatory publishing practices create further damage. These scientific outlets reward volume over quality, thus distorting evaluation criteria. They promote negative selection by favouring speed of publication at the expense of rigorous peer review. Over time, this weakens academic standards and undermines trust in the research output. The result is a decline in scientific credibility and an overall reduction in international competitiveness. Although predatory publishing is motivated by financial gain, it results in serious institutional consequences. It directly reshapes hiring, promotion, and funding decisions in ways that disadvantage high-quality research. This contributes to the erosion of both research integrity and academic communities.

In this editorial, we propose a complementary, potentially controversial, policy approach to counter “brain drain” in middle-income and transition countries. The idea is to establish competitive funding schemes that explicitly link research excellence to geographic location. For example, European Research Council (ERC) type grants, which are awarded solely based on scientific excellence, may establish a parallel track that requires the funded research to be carried out in EU Widening and associated countries^1^(“Widening countries” is a term used in European Union research policy to denote EU member states and associated countries with research and innovation performance below the EU average. The category includes most Central and Eastern European countries, as well as Portugal, Greece, and Cyprus, and is reflected in persistently lower success rates in competitive EU research funding schemes compared with higher-performing member states. Associated countries are eligible for hosting a Widening coordinator and include 14 Associated Countries with equivalent characteristics in terms of R&I performance (https://rea.ec.europa.eu/horizon-europewidening-who-should-apply_en.), many of which face structural constraints in research and innovation capacity. By opening such positions to international competition and selecting from the highest-quality researchers globally, this model attracts established scientists to work within developing research environments under ERC-type conditions, thereby effectively reversing brain drain by coupling excellence-based selection with institutional capacity building in the host country.

## Basic research and long-term scientific capacity

It is well-established that basic research supports long-term scientific and technological capacity by generating foundational knowledge. The effects are often indirect and delayed; however, translation to applied science almost always depends on prior fundamental work (Petit 2004, Ribeiro 2021). Breakthroughs in applied research, industry solutions, and innovations rooted in real-world problems are almost always underpinned by earlier investments in basic science (Welsch 2021). Sustained support for fundamental research thus inevitably leads to strong national research systems and reduces long-term reliance on imported technologies and expertise.

Strong basic research capacity improves a country’s ability to manage technological change, respond to crises, and engage in international collaboration. In addition, basic research also supports local problem-solving capacity and contributes to retaining skilled researchers. Where such research environments exist, they offer stable working conditions and career paths that make long-term employment more viable for researchers. In contrast, neglecting basic research limits countries that are forced to import knowledge and know-how rather than generating it. This, in turn, reinforces their dependence on foreign innovation.

Despite these considerations, science policy in many countries with limited research funding treats basic research as non-essential. Throughout this article, we will use the term “under-resourced countries” to denote countries with limited research and innovation capacity, including middle-income and transition economies, as well as several EU Widening and associated countries. These countries are not necessarily economically under-resourced by global standards but often face structural constraints in their research systems, including limited institutional capacity, weaker funding frameworks, and persistent loss of scientific talent.

Policymakers often prioritize applied or translational research because of limited resources and often unrealistic expectations of short-term returns, unfortunately forgetting that, over time, the viability of every applied research ultimately depends on the presence of a strong foundational research base. It must be pointed out, however, that historical experience shows that the emergence of strong national research systems is a gradual process that unfolds over decades (Etzkowitz and Leydesdorff 2000). While financial investment in research infrastructure and funding programs is essential, resources alone are insufficient to create a durable scientific ecosystem. Successful research systems depend on the progressive development of institutional stability, transparent evaluation practices, academic freedom, and a culture of inquiry that supports critical thinking and long-term discovery. These elements typically evolve slowly through the accumulation of scientific expertise, mentorship traditions, and stable governance structures within universities and research institutes.

These policy choices in turn contribute to persistent differences in national research capacity, which cannot be explained by funding levels alone. Limited human capital, weak research environments, and poorly structured research systems and incentives further deteriorate research output and quality. These factors interact with economic constraints to form a self-reinforcing, escalating loop involving limited research capacity, constrained social development, and low economic productivity (Badr 2018a, Lee 2022).

## Brain drain and institutional decline in academic research

Administrative reforms alone are insufficient to raise educational attainment; durable improvements depend on investments in instructional quality, faculty development, and institutional support structures. Intellectual capacity in academia accumulates over decades and depends on institutional continuity rather than short-term reforms (Vose and Cervellini 1983). One of the main drivers of poor research performance, especially in middle-income and transition countries, including EU Widening countries, is the loss of skilled researchers through sustained emigration, the so-called “brain drain” (Batista et al. 2025).

In addition to removing experienced and highly productive scientists from domestic institutions (Weinberg 2011), brain drain also alters incentives for the scientists who remain. As career prospects become more limited, research activity often shifts toward promotion- or income-driven work rather than long-term research agendas. In addition, universities facing staff shortages lower recruitment standards, which further reduces research quality and weakens academic institutions. Over time, this process promotes institutional decline.

Temporary mobility, paired with strong incentives, represents one of the fastest ways to transmit research norms and practices. Unfortunately, researchers who return after working abroad often encounter rigid administrative structures and limited research autonomy. In many cases, they are unable to apply the skills and practices that they have acquired abroad. Ironically, rather than strengthening institutions, their reintegration is frequently constrained by the same conditions that motivated their departure (Badr 2018a,b, 2019).

One of the most serious academic problems in many EU Widening and associated countries is the sustained emigration of highly qualified researchers which poses a structural risk to universities and public research institutions (El Saghir et al. 2020). Human capital loss reduces scientific capacity and weakens the institutional foundations required for long-term innovation. The effects extend beyond individual departures. Without a critical mass of capable researchers, universities struggle to maintain academic standards, develop coherent research agendas, or implement effective science policies. Under favourable institutional conditions, outward migration may contribute to ‘brain circulation’, whereby researchers who trained or worked abroad later return to their home countries or maintain sustained scientific collaborations that facilitate knowledge transfer and capacity building. Historical examples demonstrate that large-scale return migration can contribute substantially to the development of national research systems. However, such positive effects depend on the presence of stable research environments, credible career opportunities, and institutional structures capable of integrating returning scientists. In the absence of these conditions, sustained outward migration more often leads to a persistent loss of scientific capacity rather than to productive circulation of talent.

As institutional capacity declines due to brain drain, formal strategies for scientific development often remain symbolic. Policies are adopted but not executed, and expected outcomes often fail to materialize. Addressing these conditions requires mechanisms that strengthen researchers’ retention, establish credible career structures, and restore merit-based evaluation. One underexplored approach that we here propose is a funding mechanism to reverse brain drain through the use of geographically constrained excellence-based funding tracks. In such a scenario, highly competitive grants are awarded solely on scientific quality but with one caveat: they require the funded research be carried out within a designated under-resourced country. By linking international competition to local institutional embedding, such schemes elegantly invert traditional patterns of brain drain, attracting highly qualified researchers into rather than out of these systems.

## Infrastructure and funding constraints in research institutions

Beyond human capital, limitations in physical research infrastructure also impose structural constraints on scientific activity by narrowing the range of research problems institutions can credibly pursue. Persistent deficits in shared facilities and long-term infrastructure financing prevent many institutions from sustaining research programs that meet international standards, regardless of the quality of individual researchers. Thus, institutional competitiveness depends not only on scientific talent but also on the access to stable and well-functioning, collective research infrastructure (https://www.esfri.eu/esfri-report-funding-research-infrastructures).

At the same time, a paradoxical situation can also be commonly observed. Substantial investments are often made in advanced equipment, but without a clear scientific rationale for their use and/or without researchers being capable of using it effectively. This mismatch is observed not only in middle-income and transition countries but also in several newer EU member states and widening countries, where research and innovation performance remains below the EU average. In these cases, structural and cohesion funds are frequently directed toward infrastructure without parallel investment in recruitment, training, or retention of researchers (https://www.eua.eu/our-work/expert-voices/to-bridge-europes-r-i-gap-we-first-need-to-change-the-conversation-on-widening.html). As a result, expensive equipment is often underused or becomes obsolete before it can be integrated into productive research programs. This reflects shortcomings in national planning but also weaknesses in external funding oversight. Infrastructure funding decisions are, unfortunately, frequently insufficiently linked to assessments of scientific needs, institutional capacity, or long-term sustainability.

Researchers in these settings also face unfavourable procurement conditions. Scientific equipment, reagents, and research services are often priced substantially higher than in higher-income countries, despite limited justifications for these differences (Jonjic and Traven 2004). Suppliers commonly cite small market size, but such explanations do not account for the scale of observed price disparities. When combined with complex procurement procedures and administrative delays, these conditions disrupt research workflows and, in some cases, make experimental work extremely hard to carry out (Badr 2018b; Vose 1983).

Funding levels further constrain research activity. World Bank data indicate that developing countries invest, on average, 0.66% of their GDP in research and development, compared with a global average of 2.64%. High-income countries, on the other hand, allocate approximately 2.9% of GDP, while low- and middle-income countries invest around 1.9% (https://data.worldbank.org/indicator/GB.XPD.RSDV.GD.ZS). In many cases, available grants are insufficient to support projects beyond their initial scope.

Allocation practices further reduce effectiveness. Limited subject-matter expertise among reviewers and uneven distribution of funds among reviewing boards and panels often contribute to the rejection of strong grant proposals and support of weaker ones. These national-level issues strongly affect institutional preparedness for competitive EU grants, influencing proposal quality and submission rates, thereby translating domestic capacity limitations into persistently low success rates at the European level.

## Research culture, evaluation practices, and predatory publishing

Beyond funding constraints and staffing shortages, research capacity is also shaped by academic culture and evaluation practices. In many countries, career advancement depends more on publication counts than on the substance of research contributions (Joynson and Leyser 2015, Demir 2018). Hiring, promotion, and degree requirements as a rule, rely on formal metrics rather than expert assessment of scientific content (Timmis et al. 2025, Trueblood et al. 2025).

This emphasis on quantity creates incentives to prioritize rapid publication over rigorous research. Evaluation systems often rely on journal-level indicators, publication counts, and citation metrics, while giving limited weight to reproducibility, methodological soundness, or originality. As a result, researchers are incentivized to prioritize publication volume over substantive novelty (Kashyap et al. 2024, Öztürk and Taskin 2024). This environment selects for journals and other scientific outlets that apply a weak peer review process and often rely on unclear or non-transparent editorial procedures (Öztürk and Taskin 2024).

To add insult to injury, researchers who adhere to stricter scientific standards face structural disadvantages. Limited access to advanced infrastructure and external research services constrains their ability to meet the expectations of high-impact international journals. In competitive evaluation systems that reward output volume, such researchers may publish less frequently and, as a consequence, fall behind in career advancement. Some eventually turn to lower-quality or less transparent journals to remain competitive, even while aware of the long-term costs to their scientific credibility and funding prospects.

Funding agencies often reinforce these incentives. Short project timelines and rigid output requirements fail to account for research areas that require extended validation of results or complex experimentation. Pressure to produce publications early can reduce research quality and lead to inefficient use of limited resources. Similar patterns have been documented across multiple national contexts (Moher et al. 2017, Demir 2018).

Time pressure and administrative workload further weaken academia. Senior researchers, due to these issues, often lack the capacity to provide sustained guidance to early-career scientists. When administrative load couples with a strong publication pressure, unethical practices and reliance on predatory journals become much more likely (de Lorenzo et al. 2025, Byrne 2026). Empirical analyses indicate that a large share of articles published in predatory journals originate from lower- and middle-income countries, with reported proportions exceeding 80% in some datasets (Moher et al. 2017, Demir 2018, Koçak 2022).

In parallel, some governments provide direct financial rewards per publication without regard to journal quality. These incentive structures legitimize predatory publishing and reinforce metric-driven behaviour. As observed in analyses of research incentives, misaligned administrative and evaluation frameworks encourage output maximization at the expense of research integrity and long-term value (Koçak 2022). Early-career researchers internalize these practices through observation, reinforcing a culture that prioritizes publication counts over scientific contribution. The expansion of predatory publishing thus causes systemic and serious harm through distorted evaluation and selection. It is not a marginal nor an ethical issue but a structural and systemic problem with serious consequences. Predatory practices weaken assessment standards, erode research credibility, and undermine trust in scientific outputs. In settings with limited resources and fragile institutions, these effects are additionally amplified. Improving research quality under such conditions requires aligning incentives with excellence through transparent evaluation criteria, merit-based career progression, selection mechanisms that reward rigor rather than volume, as well as fostering prestige-linked competition and researchers’ autonomy. Without these reforms, increases in publication output are unlikely to translate into long-term scientific capacity or international credibility (Timmis et al. 2025).

## Persistent research disparities within the european union

Differences in research capacity are not limited to comparisons between different regions of the world. They are also pronounced within the European Union itself (Molica and Santos 2025). Researchers based in widening and associated countries continue to lag behind those in longer-established EU member states in access to resources and in success rates in competitive funding schemes, most notably European Research Council (ERC) grants (Naujokaitytė 2025). Although recent data show some incremental improvement, the gap remains substantial.

The causes of this disparity are multifactorial (Auditors 2022). While underfunding is frequently cited, it explains only part of the divergence. EU enlargement was expected to accelerate scientific development in accession countries, yet despite measurable progress, relative competitiveness has improved slowly. This experience illustrates the difficulty of building research excellence within short time frames, even when supranational instruments are explicitly designed to support convergence.

Several widening countries have implemented extensive national reforms, including new funding agencies and evaluation frameworks. However, outcomes have consistently fallen short of expectations. As a result, research performance in newer EU member states remains well below that of older members. The imbalance is clearly reflected in ERC funding outcomes: the fifteen newer EU member states together secured €381.7 million out of a total €7.3 billion in ERC funding according to a recent report (Naujokaitytė 2025). These figures indicate that existing instruments have not substantially altered the competitive landscape.

Although many widening countries have increased public investment in science, funding growth has not translated into an improved long-term research capacity (Fifeková et al. 2021). Research institutions often remain vulnerable to political intervention, shifting priorities, and unstable governance structures. Early gains are therefore difficult to sustain, and progress frequently plateaus. In this context, additional funding alone cannot compensate for institutional weaknesses that limit excellence-driven research.

The European Union has introduced multiple instruments to address these disparities, yet their aggregate impact has been modest, at best (Veugelers 2016, Maris 2024). It remains unclear whether limited effectiveness reflects weaknesses in the design and evaluation of hybrid funding schemes or structural constraints within recipient countries. ERC performance remains the most reliable indicator of this lag, as it applies uniform excellence criteria across the EU (Ghirelli et al. 2023).

Proposals to correct the imbalance through special instruments for scientifically less-developed countries have surfaced repeatedly but have not been implemented. A more promising approach would involve ERC calls specifically tailored to EU Widening and associated countries with lower research & innovation capacity, while maintaining identical evaluation standards and full international openness. In this model, ERC-type grants, awarded solely on the basis of scientific excellence, would operate through a parallel track requiring the funded research to be carried out in a designated widening or associated country. By opening these positions to international competition and selecting from the highest-quality researchers globally, such schemes attract established scientists into developing research environments. In doing so, as already stated, they effectively reverse traditional patterns of brain drain.

The principal advantage of this approach is that it builds research capacity through the sustained presence of highly productive investigators. Hosting projects led by outstanding scientists would strengthen local research environments and train doctoral and postdoctoral researchers who could later compete successfully in EU-wide funding schemes. This mechanism would also help retain talented early-career scientists. Additionally, granting researchers the freedom to choose and, if necessary, change host institutions would foster competition for talent, creating powerful mechanisms and competitive pressure on institutions to improve working conditions, governance, and the credibility of their commitments. In the absence of policy instruments that explicitly embed high-quality research within widening countries, existing disparities in research capacity are likely to persist or deepen over time.

Historically, many of today’s strongest research systems have expanded their capacity by attracting and integrating highly trained scientists educated in less–developed settings, with empirical evidence showing that such inflows of researchers from developing countries have the capacity to significantly boost the scientific performance of host institutions and countries (Fassio et al. 2019, Capoani et al. 2024). The EU is already experimenting with similar mobility–based approaches, but with limited success. Together, the development of internationally competitive research systems is a long-term process that typically unfolds over decades and depends on sustained institutional continuity, stable funding, and the gradual accumulation of human capital. Short-term indicators provide only a partial view of national scientific capacity and should not be interpreted as a comprehensive measure of a country’s long-term scientific potential.

## Recommended actions

Research capacity does not improve through funding increases alone. It depends on institutional incentives, evaluation practices, and the ability to retain skilled researchers. Brain drain, predatory publishing, and metric-driven assessment reinforce one another and weaken research institutions over time. Addressing these problems requires coordinated institutional reform rather than isolated interventions.

Effective responses must align funding, evaluation, and career structures with research quality. Without such alignment, additional investment is unlikely to produce durable gains.

We propose these conditions can be addressed through the following measures:

- **Establish long-term grants awarded to individual principal investigators on the basis of scientific excellence, with funding anchored to EU Widening and associated countries facing lower levels of research and innovation capacity**. By this mechanism, institutions would be able to attract and retain outstanding investigators by providing credible career prospects, guaranteed research autonomy, adequate infrastructure, and dedicated administrative support.
- **Retention of scientific talent**. Establish stable research careers through transparent promotion criteria, predictable funding, and viable long-term positions. Investment in infrastructure should be matched by sustained investment in researchers.
- **Reform of evaluation and funding practices**. Reduce reliance on publication counts and journal-based metrics. Funding decisions should prioritize scientific content, rigor, and originality, and be administered by independent, expert-led bodies.
- **Moving evaluation systems away from reliance on publication counts and journal-level indicators such as impact factors**. Assessment should focus on the scientific content, methodological rigor, and originality of the work.
- **Strengthening mentorship and research integrity**. Motivate and train senior researchers for mentoring early-career scientists. Training should emphasize research ethics, methodological rigor, and awareness of the consequences of poor scientific practices.
- **Reduction of administrative burdens**. Simplify employment and funding rules that restrict mobility, delay procurement, or block merit-based advancement.
- **Support for collaboration**. Encourage international and interdisciplinary collaboration to reduce isolation, share infrastructure costs, and improve research quality.

**Sustained investment in basic research**. Applied research depends on a stable base of fundamental research. Applied scientific solutions necessitate a consistent support of basic science.

## Conclusions

In many middle-income and transition countries, weak research performance reflects institutional design and incentive structures rather than a simple lack of resources. Persistent brain drain, metric-driven evaluation, and the spread of predatory publishing stem from common underlying conditions: insecure academic career paths, promotion systems based on publication counts rather than research quality and administrative frameworks that reward short-term output over sustained, rigorous and high-quality scientific work. These conditions discourage long-term research agendas and push capable researchers to leave, undermining universities and public research institutes even where funding levels and physical infrastructure have improved. The main mechanism to counteract these problems proposed in this editorial is the use of geographically constrained, excellence-based funding instruments, such as ERC-type grants, that require funded research to be carried out within selected countries (middle-income, transition, widening, associated etc.) while maintaining identical evaluation standards and full international openness. By awarding long-term funding to individual principal investigators solely on the basis of scientific quality, and by anchoring these grants to lagging countries and institutions, such schemes offer the possibility to directly reverse brain drain into “brain gain”. Such practice has the capability of attracting established researchers, create stable research teams, and transmit high standards of evaluation, mentoring, and research practice to lagging institutions and countries. Without targeted instruments that connect international competition with local institutions and governance structures, additional investments are unlikely to translate into long-term research capacity and/or sustained international competitiveness.
